# The origins of the trypanosome genome strains *Trypanosoma brucei brucei *TREU 927, *T. b. gambiense *DAL 972, *T. vivax *Y486 and *T. congolense *IL3000

**DOI:** 10.1186/1756-3305-5-71

**Published:** 2012-04-07

**Authors:** Wendy Gibson

**Affiliations:** 1 School of Biological Sciences, University of Bristol, Bristol BS8 1UG, UK

**Keywords:** Trypanosomes, Pathogen, Genome

## Abstract

The genomes of several tsetse-transmitted African trypanosomes (*Trypanosoma brucei brucei, T. b. gambiense, T. vivax, T. congolense*) have been sequenced and are available to search online. The trypanosome strains chosen for the genome sequencing projects were selected because they had been well characterised in the laboratory, but all were isolated several decades ago. The purpose of this short review is to provide some background information on the origins and biological characterisation of these strains as a source of reference for future users of the genome data. With high throughput sequencing of many more trypanosome genomes in prospect, it is important to understand the phylogenetic relationships of the genome strains.

## Review

The genome sequence of *Trypanosoma brucei brucei *TREU 927/4 was published in 2005 [1] and that of *T. b. gambiense *Dal 972 clone 1 in 2010 [2]. Genome sequencing projects for *T. vivax *Y486 and *T. congolense *IL3000 are also complete [3]. The trypanosome strains chosen for sequencing were selected because they had been well characterised in the laboratory, but all were isolated several decades ago (Table 1). The purpose of this short review is to provide some background information on the origins and biological characterisation of these strains as a source of reference for future users of the genome data. The history of the discovery of these trypanosome species has been recently reviewed [4].

**Table 1:** Trypanosome genome strains

Species	Strain	Origin
*T. b. brucei*	TREU 927/4	Isolated from tsetse *Glossina pallidipes *in 1970 in Kiboko, Kenya
*T. b. gambiense*	DAL 972	Isolated from a patient in 1986 in Daloa, Ivory Coast
*T. vivax*	Y486	Isolated from a bovine in 1976 in Zaria, Nigeria
*T. congolense*	IL3000	Isolated from a bovine in 1966 in Transmara, Kenya

### Trypanosoma brucei brucei TREU 927/4

#### Isolation and phenotype

*Trypanosoma brucei brucei *clone TREU 927/4 was chosen as the representative *T. brucei *for the genome project, because it displays the full range of known phenotypes for *T. brucei*, barring human infectivity. TREU 927/4 is capable of complete cyclical development within the tsetse fly, including mating [5] and produces short stumpy forms during bloodstream infection in the mammalian host [6]. TREU 927/4 is a clone derived from the isolate GPAL/KE/70/EATRO 1534 [5] that originates from Kiboko, Kenya, an area where human trypanosomiasis is unknown [7]. Nevertheless, there is some doubt about the status of TREU 927/4 with regard to human infectivity, since it has a degree of resistance to human serum [8], though it lacks the *SRA *gene that is characteristic of the human infective subspecies *T. b. rhodesiense *from East Africa [9].

The isolate GPAL/KE/70/EATRO 1534 was one of a collection of 15 *T. brucei *subgroup isolates obtained from wild caught tsetse flies of the species *Glossina pallidipes *from Kiboko, Kenya [7,10,11]. Each isolate was derived from the metacyclic population of a single infected fly by inoculation of macerated salivary glands into rodents; the bloodstream forms were subsequently used to study the antigenic types circulating in wild-caught flies [11]. Like other pleomorphic *T. b. brucei *isolates, TREU 927/4 is easily grown as bloodstream forms in rodents or procyclics *in vitro*; it has also been adapted to the bloodstream form in *in vitro *culture [6] and has been transmitted via *G. morsitans morsitans *and *G. pallidipes *in the laboratory.

#### Relationship to other *T. brucei *strains

TREU 927/4, together with other *T. b. brucei *isolates from Kiboko, has been characterised by various molecular methods in a number of different studies. The consensus from these analyses is that TREU 927/4 represents a subgroup of *T. b. brucei *that is widespread in East Africa.

Initial studies of isoenzyme variation showed that the Kiboko isolates had a high frequency of rare polymorphisms for several enzymes, notably threonine dehydrogenase (TDH) and malate dehydrogenase (MDH), and that these unusual isoenzyme patterns were shared by *T. brucei *subgroup isolates from other areas with abundant wild animals in Kenya (Kibwezi, Meru and Maasai Mara), Zambia (Luangwa valley) and Tanzania (Serengeti) [12-14]. As a consequence, these isolates appeared as a separate clade distinct from other *T. brucei *subgroup isolates in cluster analysis [12-14]. The clade was initially dubbed the *kiboko *group [12] and later split into 2 groups: *kiboko *and *kakumbi *[13]. Only a minority (estimated < 7%) of the many isolates of the *T. brucei *subgroup that have been characterised belong to the *kiboko/kakumbi *isoenzyme group [12,13]. The association with areas of abundant wildlife in East Africa suggests transmission cycles primarily involving wild mammals and tsetse. However, some *kiboko/kakumbi *strains have been isolated from domestic animals and three came from humans in the Luangwa Valley, Zambia [13].

The molecular karyotype of TREU 927/4 was deduced from chromosome-separation gels and hybridisation with a comprehensive set of gene probes [15]. The nuclear genotype has been analysed by restriction fragment length polymorphisms (RFLPs), mini- and microsatellites. Reinforcing the isoenzyme results, analysis of *T. brucei *subgroup isolates using RFLP data from repetitive DNA probes placed TREU 927/4 in a discrete cluster with 2 other Kiboko tsetse isolates [16,17]. Minisatellite analysis of the original trypanosome population from which TREU 927/4 was cloned showed it to contain several genotypes [18]. Minisatellites are hypervariable and the Kiboko strains, like other collections of trypanosome isolates from particular locations, show population specific alleles [19]. A recent study using STRUCTURE to analyse microsatellite genotype data from a total of 142 *T. brucei *subgroup strains placed TREU 927/4 in a cluster designated Kiboko B, including 18 other *T. b. brucei *isolates derived from wild animals (mostly lions and hyenas) in Serengeti, Tanzania, and other tsetse and livestock isolates from Kiboko and Meru in Kenya [20]. In summary, there is agreement from all genotyping analyses to date that TREU 927/4 belongs to a particular group of *T. b. brucei *isolates that is found predominantly in wildlife areas of East Africa.

Complementary to these data from analyses of the nuclear genome, are data on variation in the sequence of kinetoplast DNA maxicircles, which constitute the mitochondrial genome of trypanosomes. Initial analysis of RFLPs showed that the maxicircles of *kiboko/kakumbi *strains could be distinguished from those of other *T. brucei *subgroup isolates by several polymorphisms [21]. Sequencing of the maxicircle COI (cytochrome c oxidase subunit I) gene allowed detailed analysis of mitochondrial haplotypes and revealed that isolates of the *kiboko/kakumbi *group, including TREU 927/4, share a limited range of haplotypes not found among other *T. brucei *subgroup isolates [20]. There was a particularly striking correlation between maxicircle haplotypes and the corresponding microsatellite data for the cluster of 19 *T. b. brucei *isolates designated Kiboko B, which included TREU 927/4 [20]. This reinforces the distinctive nature of this group of strains and also demonstrates that genetic exchange between this group and other *T. brucei *genotypes is not sufficiently frequent to break up this association.

### Trypanosoma brucei gambiense DAL 972 clone 1

#### Isolation and phenotype

*T. b. gambiense *is the causative organism of human trypanosomiasis in West and Central Africa and has been divided into two groups or types based on phenotypic and genotypic characteristics [22]. Most isolates belong to Tbg1, which conforms to the classical description of *T. b. gambiense *as a pathogen that causes a chronic disease in human patients and manifests very low parasitaemia; it is typically slow growing in experimental rodents and is better adapted to transmission by *palpalis *than *morsitans *group tsetse flies [23]. Tbg1 is responsible for most cases of human trypanosomiasis in Africa. By contrast Tbg2 grows well in experimental rodents and is easily transmitted by *morsitans *group tsetse [23]; it has rarely been isolated from patients and has thus far been found only in Côte d'Ivoire (Ivory Coast) and Burkina Faso (Upper Volta) [16,24]. Tbg2 isolates are genetically heterogeneous [16,24,25].

DAL 972 (MHOM/CI/86/DAL972) clone 1 was chosen as the representative Tbg1 for the genome project, because it was originally isolated from a patient in West Africa (Daloa focus in Côte d'Ivoire) [26], and has the phenotypic features typical of Tbg1. DAL 972 clone 1 has been very little passaged and has an extremely chronic phenotype in normal experimental rodents, but can be grown in immunosuppressed or immunodeficient mice such as SCID (severe combined immunodeficiency) mice. It has been transmitted via *G. palpalis gambiensis, G. m. morsitans *and *G. pallidipes *in the laboratory, and grows satisfactorily as procyclics *in vitro *in Cunningham's medium.

#### Relationship to other *T. brucei *strains

By biochemical characterisation, DAL 972 is a typical Tbg1 with specific isoenzyme patterns for alanine and aspartate aminotransferases [27], and has all three characteristic Tbg1 genetic markers, namely genes for the variant surface glycoproteins LiTat 1.3 and VSG AnTat 11.17, and the flagellar pocket glycoprotein, TgsGP [28,29]. Note that *TgsGP *is not correctly annotated in the DAL 972 genome, because this gene has been subject to a rearrangement relative to TREU 927 resulting in lack of synteny for one homologue of chromosome 2 [30]. In addition, DAL 972 was shown to have the characteristic Tbg1 RFLP pattern for *VSG 117 *(*AnTat 1.8*) [31,32].

Tbg1 isolates typically have low DNA contents compared to other members of the *T. brucei *subgroup [33,34]. The DNA content of DAL 972 was at the high end of the range for Tbg1, but lower than *T. b. brucei *or *T. b. rhodesiense *[34]. In addition, the molecular karyotypes of Tbg1 isolates typically have lower numbers of minichromosomes than *T. b. brucei *or *T. b. rhodesiense *[33] and this is also true for DAL 972 [34].

### Trypanosoma vivax Y486

#### Isolation and phenotype

*T. vivax *is a major pathogen of ruminants both in Africa, where it is transmitted cyclically by tsetse flies, and in South America, where it is transmitted mechanically by biting flies such as tabanids. Despite its importance as a livestock pathogen, *T. vivax *has received little attention because it is difficult to cultivate in the laboratory. Unlike *T. brucei, T. vivax *is typically not infective to laboratory rodents, but can be gradually adapted to these hosts by for example co-injection with ruminant serum or immunosuppression by sublethal irradiation [35]. However, three strains of *T. vivax *(Y486, Y58, V953) from naturally infected cattle in Zaria, Nigeria were isolated directly into mice [36]. These spontaneously mouse infective strains grow to high parasitaemia in outbred mice and have been widely used in laboratory studies. The ability to clone antigenic variants of *T. vivax *Y486 [37] led to the purification and characterization of *T. vivax *variant surface glycoproteins [38]; *T. vivax *Y486 is ILRAD *Duttonella *antigen repertoire 1, ILDAR 1. As the most well characterized of the three Zaria *T. vivax *strains, Y486 was chosen for the genome project.

The Zaria *T. vivax *strains are infective to calves, sheep and goats, as well as mice, rats and rabbits, and are readily transmissible by a wide range of tsetse species (*G. m. centralis, G. m. morsitans, G. pallidipes, G. austeni, G. brevipalpis, G. tachinoides, G. palpalis palpalis, G. p. gambiensis, G. fuscipes fuscipes*) [37,39-42]. Infection in these flies was typically found in the labrum and hypopharynx and sometimes also in the cibarium [41,42]. Unlike *T. brucei *and *T. congolense, T. vivax *does not multiply in the tsetse midgut and hence does not grow *in vitro *as procyclics. Although culture systems to maintain *T. vivax *bloodstream forms *in vitro *were developed by several groups [43-45], none has come into general use. Likewise, there are several published methods from the same era for *in vitro *differentiation of bloodstream forms into epimastigotes and subsequent development to infective metacyclics [46-48]. Recently, a simple *in vitro *cultivation method for epimastigotes of IL 1392, a derivative of Y486, has been described that is robust enough to allow transfection and also gives rise to infective metacyclics [49].

#### Relationship to other *T. vivax *strains

The Nigerian reference isolate, *T. vivax *Y486, is a representative of the West African form of *T. vivax*. Before the advent of the Polymerase chain reaction (PCR), knowledge of genetic diversity in *T. vivax *was severely limited because of the difficulty of obtaining enough trypanosomes for analysis. However, differences in pathogenicity were recognised between East and West African *T. vivax *strains, the West African strains being generally regarded as more pathogenic to cattle than East African strains [50], although highly pathogenic haemorhaggic *T. vivax *strains are also known in East Africa [51,52]. West African *T. vivax *strains can be identified by specific repetitive DNA sequences and are phylogenetically distinct from East African strains [53-56]. South American *T. vivax *strains have been shown to have close genetic similarity to West African strains [57], in agreement with the historical evidence that *T. vivax *was imported into the New World in cattle from West Africa [58]. Thus, *T. vivax *Y486 also represents the South American form of *T. vivax*.

### Trypanosoma congolense IL3000

#### Isolation and phenotype

IL 3000 is a derivative of strain Transmara I, which was isolated from a bovine in the Transmara region of Kenya in 1966 [59,60]. Antigenic variation has been studied in this strain (designated ILRAD *Nannomonas *antigen repertoire 2, ILNAR 2) and variant antigen genes have been characterized from both bloodstream and metacyclic IL 3000 trypanosomes [61,62]. Presumably this was one of the deciding factors in choosing IL3000 for the genome project, although several other *T. congolense *strains such as TREU 1457 have also been widely used in experimental studies.

*T. congolense *generally grows well in laboratory rodents and bloodstream forms of IL3000 have been grown in mice as well as bloodstream form culture [60,63]. *T. congolense *is transmissible by a range of tsetse species and the life cycle is similar to that of *T. brucei *with proliferation of procyclics in the midgut and transmission of metacyclics in the saliva; for *T. congolense*, production of metacyclics occurs in the proboscis [58,64]. The tsetse developmental stages of *T. congolense *are more amenable to *in vitro *culture than those of *T. brucei *and methods for the *in vitro *production of attached epimastigotes and metacyclics pioneered by Gray and colleagues [65] have allowed the analysis of epimastigote and metacyclic as well as procyclic and bloodstream form populations. In this way epimastigote and metacyclic populations of sufficient purity for EST analysis were obtained from IL3000 [66] and recently the complete life cycle of IL3000 has been reproduced *in vitro *[63]. The ability to culture epimastigotes of IL3000 led to the characterisation of an epimastigote-specific, GPI-anchored surface glycoprotein called CESP [67], adding to the list of surface molecules specific to tsetse developmental forms of *T. congolense*, including GARP (glutamic acid/alanine rich protein) [68,69], PRS (protease-resistant surface molecule) [70] and *T. congolense *procyclin [71]. It is possible to transfect *T. congolense *procyclics fairly easily by electroporation [63,72,73], but much more difficult to transfect bloodstream forms [63].

#### Relationship to other *T. congolense *strains

Three genetically distinct subgroups are currently recognized within *T. congolense*: savannah, forest and Kenya Coast or kilifi [74]. The most abundant and widespread is *T. congolense *savannah and IL3000 represents this subgroup. The savannah and forest subgroups were originally distinguished by different isoenzyme patterns [75,76], and subsequently by unique repetitive DNA sequences which provide targets for species-specific PCR identification [77]. A further genetically distinct subgroup was isolated from livestock on the Kenya Coast [78] and appears to have a restricted distribution in East Africa. Although phylogenetic analysis places these three *T. congolense *subgroups in a single clade [79,80], they are arguably sufficiently genetically divergent to warrant recognition as separate species [81].

## Conclusions

This short review has brought together background information on the origins and biological characterisation of the four African tsetse-transmitted genome strains as a source of reference for future users of the genome data.

## Competing interests

The author declares that she has no competing interests.

## References

[B1] Berriman M Ghedin E Hertz-Fowler C Blandin G Renauld H Bartholomeu DC Lennard NJ Caler E Hamlin NE Haas B The genome of the African trypanosome *Trypanosoma brucei* Science 2005 309 416 422 10.1126/science.1112642 16020726

[B2] Jackson AP Sanders M Berry A McQuillan J Aslett MA Quail MA Chukualim B Capewell P MacLeod A Melville SE The genome sequence of *Trypanosoma brucei gambiense*, causative agent of chronic human African trypanosomiasis PLoS Negl Trop Dis 2010 4 4 e658 10.1371/journal.pntd.0000658 20404998 PMC2854126

[B3] Jackson AP Berry A Aslett M Allison HC Burton P Vavrova-Anderson J Brown R Browne H Corton N Hauser H Antigenic diversity is generated by distinct evolutionary mechanisms in African trypanosome species PNAS 2012 109 3416 3421 10.1073/pnas.1117313109 22331916 PMC3295286

[B4] Steverding D The history of African trypanosomiasis Parasites and Vectors 2008 1 3 10.1186/1756-3305-1-3 18275594 PMC2270819

[B5] Turner CMR Sternberg J Buchanan N Smith E Hide G Tait A Evidence that the mechanism of gene exchange in *Trypanosoma brucei *involves meiosis and syngamy Parasitology 1990 101 377 386 10.1017/S0031182000060571 1982633

[B6] van Deursen FJ Shahi SK Turner CMR Hartmann C Guerra-Giraldez C Matthews KR Clayton CE Characterisation of the growth and differentiation in vivo and in vitro of bloodstream form *Trypanosoma brucei *strain TREU 927 Mol Biochem Parasitol 2001 112 163 171 10.1016/S0166-6851(00)00359-5 11223123

[B7] Goedbloed E Ligthart G Minter D A comparison of *Trypanosoma brucei *subgroup isolates from *Glossina pallidipes *in two areas of Kenya Trans Roy Soc Trop Med Hyg 1971 65 261 263 10.1016/0035-9203(71)90272-0

[B8] Turner CMR McLellan S Lindergard LAG Bisoni L Tait A MacLeod A Human infectivity trait in *Trypanosoma brucei*: stability, heritability and relationship to *sra *expression Parasitology 2004 129 445 454 10.1017/S0031182004005906 15521633

[B9] Radwanska M Chamekh M Vanhamme L Claes F Magez S Magnus E De Baetselier P Buscher P Pays E The serum resistance-associated gene as a diagnostic tool for the detection of *Trypanosoma brucei rhodesiense* Am J Trop Med Hyg 2002 67 684 690 12518862 10.4269/ajtmh.2002.67.684

[B10] Dar FK Wilson AJ Goedbloed E Ligthart GS Minter D Serological studies on trypanosomiasis in East Africa. I: introduction and techniques Ann Trop Med Parasit 1973 67 21 29 4723212 10.1080/00034983.1973.11686860

[B11] Goedbloed E Ligthart GS Minter DM Wilson AJ Dar FK Paris J Serological studies on trypanosomiasis in East Africa. II: Comparisons of antigenic types of *Trypanosoma brucei *subgroup organisms isolated from wild tsetse flies Ann Trop Med Parasit 1973 67 31 43 4723213

[B12] Gibson WC Marshall TFdC Godfrey DG Numerical analysis of enzyme polymorphism: a new approach to the epidemiology and taxonomy of trypanosomes of the subgenus *Trypanozoon* Adv Parasitol 1980 18 175 246 7001872 10.1016/s0065-308x(08)60400-5

[B13] Godfrey DG Baker RD Rickman LR Mehlitz D The distribution, relationships and identification of enzymic variants within the subgenus *Trypanozoon* Adv Parasitol 1990 29 1 74 2181826 10.1016/s0065-308x(08)60104-9

[B14] Mathieu-Daude F Tibayrenc M Isozyme variability of *Trypanosoma brucei *s.l.: genetic, taxonomic, and epidemiological significance Expt Para 1994 78 1 19 10.1006/expr.1994.1001 8299755

[B15] Melville SE Leech V Gerrard CS Tait A Blackwell JM The molecular karyotype of the megabase chromosomes of *Trypanosoma brucei *and the assignment of chromosome markers Mol Biochem Parasitol 1998 94 155 173 10.1016/S0166-6851(98)00054-1 9747967

[B16] Hide G Cattand P Le Ray D Barry DJ Tait A The identification of *Trypanosoma brucei *subspecies using repetitive DNA sequences Mol Biochem Parasitol 1990 39 213 226 10.1016/0166-6851(90)90060-Y 1969612

[B17] Hide G Buchanan N Welburn S Maudlin I Barry JD Tait A *Trypanosoma brucei rhodesiense*: characterisation of stocks from Zambia, Kenya, and Uganda using repetitive DNA probes Expt Para 1991 72 430 439 10.1016/0014-4894(91)90089-F 2026217

[B18] MacLeod A Turner CMR Tait A A high level of mixed *Trypanosoma brucei *infections in tsetse flies detected by three hypervariable minisatellites Mol Biochem Parasitol 1999 102 237 248 10.1016/S0166-6851(99)00101-2 10498180

[B19] MacLeod A Turner CMR Tait A The detection of geographical substructuring of *Trypanosoma brucei *populations by the analysis of minisatellite polymorphisms Parasitology 2001 123 475 482 11719958 10.1017/s0031182001008666

[B20] Balmer O Beadell JS Gibson W Caccone A Phylogeography and taxonomy of *Trypanosoma brucei* PLoS NTD 2011 5 e961 10.1371/journal.pntd.0000961 PMC3035665 21347445

[B21] Gibson WC Fase-Fowler F Borst P Further analysis of intraspecific variation in *Trypanosoma brucei *using restriction site polymorphisms in the maxi-circle of kinetoplast DNA Mol Biochem Parasitol 1985 15 21 36 10.1016/0166-6851(85)90026-X 2985985

[B22] Gibson WC Will the real *Trypanosoma brucei gambiense *please stand up? Parasitol Today 1986 2 255 257 10.1016/0169-4758(86)90011-6 15462856

[B23] Richner D Brun R Jenni L Production of metacyclic forms by cyclical transmission of West African *Trypanosoma (Trypanozoon) brucei *isolates from man and animals Acta Trop 1988 45 309 319 2467539

[B24] Mehlitz D Zillmann U Scott CM Godfrey DG Epidemiological studies on the animal reservoir of gambiense sleeping sickness. III. Characterisation of *Trypanozoon *stocks by isoenzymes and sensitivity to human serum Tropenmed Parasit 1982 33 113 118 6287687

[B25] Biteau N Bringaud F Gibson W Truc P Baltz T Characterization of *Trypanozoon *isolates using a repeated coding sequence and microsatellite markers Mol Biochem Parasitol 2000 105 185 201 10693742

[B26] Dukes P Kaukus A Hudson KM Asonganyi T Gashumba JK A new method for isolating *Trypanosoma brucei gambiense *from sleeping sickness patients Trans Roy Soc Trop Med Hyg 1989 83 636 639 10.1016/0035-9203(89)90379-9 2617625

[B27] Stevens JR Lanham SM Allingham R Gashumba JK A simplified method for identifying subspecies and strain groups in *Trypanozoon *by isoenzymes Ann Trop Med Parasit 1992 86 9 28 1616401 10.1080/00034983.1992.11812626

[B28] Bromidge T Gibson W Hudson KM Dukes P Identification of *Trypanosoma brucei gambiense *by PCR amplification of variant surface glycoprotein genes Acta Trop 1993 53 107 119 10.1016/0001-706X(93)90023-5 8098897

[B29] Gibson W Nemetschke L Ndung'u J Conserved sequence of the TgsGP gene in Group 1 *Trypanosoma brucei gambiense* Infect Genet Evol 2010 10 453 458 10.1016/j.meegid.2010.03.005 20302972

[B30] Felu C Pasture J Pays E Perez-Morga D Diagnostic potential of a conserved genomic rearrangement in the *Trypanosoma brucei gambiense*-specific TGSGP locus Am J Trop Med Hyg 2007 76 922 929 17488917

[B31] Dukes P Gibson WC Gashumba JK Hudson KM Bromidge TJ Kaukus A Assonganyi T Magnus E Absence of the LiTat 1.3 (CATT antigen) gene in *Trypanosoma brucei gambiense *stocks from Cameroon Acta Trop 1992 51 123 134 10.1016/0001-706X(92)90054-2 1354930

[B32] Kanmogne GD Stevens JR Asonganyi T Gibson WC Characterisation of *Trypanosoma brucei gambiense *isolates using restriction fragment length polymorphisms in 5 variant surface glycoprotein genes Acta Trop 1996 61 239 254 10.1016/0001-706X(96)00006-X 8790774

[B33] Dero B Zampetti-Bosseler F Pays E Steinert M The genome and the antigen gene repertoire of *Trypanosoma brucei gambiense *are smaller than those of *T. b. brucei* Mol Biochem Parasitol 1987 26 247 256 10.1016/0166-6851(87)90077-6 3431572

[B34] Kanmogne GD Bailey M Gibson W Wide variation in DNA content among isolates of *Trypanosoma brucei *ssp Acta Trop 1997 63 75 87 10.1016/S0001-706X(96)00600-6 9088421

[B35] Gathuo HKW Nantulya VM Gardiner PR *Trypanosoma vivax*: adaptation of two East African stocks to laboratory rodents J Protozoo 1987 34 48 53 10.1111/j.1550-7408.1987.tb03130.x 3572841

[B36] Leeflang P Buys J Blotkamp C Studies on *Trypanosoma vivax *- Infectivity and serial maintenance of natural bovine isolates in mice Int J Parasitol 1976 6 413 417 10.1016/0020-7519(76)90027-8 965146

[B37] Barry JD Antigenic variation during *Trypanosoma vivax *infections of different host species Parasitology 1986 92 51 65 10.1017/S0031182000063447 2421230

[B38] Gardiner PR Pearson TW Clarke MW Mutharia LM Identification and isolation of a variant surface glycoprotein from *Trypanosoma vivax* Science 1987 235 774 777 10.1126/science.3810164 3810164

[B39] De Gee ALW Ige K Leeflang P Studies on *Trypanosoma vivax*: Transmission of mouse infective *T. vivax *by tsetse flies Int J Parasitol 1976 6 419 421 10.1016/0020-7519(76)90028-X 965147

[B40] Moloo SK Sabwa CL Kabata JM Vector competence of *Glossina pallidipes *and *Glossina morsitans centralis *for *Trypanosoma vivax, Trypanosoma congolense *and *T. b. brucei* Acta Trop 1992 51 271 280 10.1016/0001-706X(92)90045-Y 1359753

[B41] Moloo SK Kutuza SB Desai J Comparative study on the infection rates of different *Glossina *species for East African and West African *Trypanosoma vivax *stocks Parasitology 1987 95 537 542 10.1017/S0031182000057966 3696778

[B42] Jefferies D Helfrich MP Molyneux DH Cibarial infections of *Trypanosoma vivax *and *T. congolense *in *Glossina* Parasitol Res 1987 73 289 292 10.1007/BF00531079 3615393

[B43] Zweygarth E Gray MA Kaminsky R Axenic in vitro cultivation of *Trypanosoma vivax *trypomastigote forms Trop Med Parasitol 1991 42 45 48 2052856

[B44] Brun R Moloo SK In vitro cultivation of animal infective forms of a West African *Trypanosoma vivax *stock Acta Trop 1982 39 135 141 6126095

[B45] Hirumi H Hirumi K Moloo SK Shaw MK In vitro cultivation of bloodstream trypomastigotes of *Trypanosoma vivax *without feeder layer cells J Protozoo Res 1991 1 1 12

[B46] Hirumi H Nelson RT Hirumi K Complete cyclic development of *Trypanosoma vivax in vitro* J Protozoo 1983 30 A6

[B47] Gumm ID The axenic cultivation of insect forms of *Trypanosoma (Duttonella) vivax *and development to the infective metacyclic stage J Protozoo 1991 38 163 171 10.1111/j.1550-7408.1991.tb04424.x 1880757

[B48] Stiles JK Wallbanks KR Molyneux DH Metacyclogenesis of *Trypanosoma vivax *in vitro - attachment to chitosan gel Ann Trop Med Parasit 1990 84 197 200 2383099 10.1080/00034983.1990.11812456

[B49] D'Archivio S Medina M Cosson A Chamond N Rotureau B Minoprio P Goyard S Genetic engineering of *Trypanosoma (Duttonella) vivax *and *in vitro *differentiation under axenic conditions PLoS NTD 2011 5 e1461 10.1371/journal.pntd.0001461 PMC3246432 22216367

[B50] Stephen LE Trypanosomiasis, a veterinary perspective 1986 Oxford: Pergamon Press

[B51] Gardiner PR Assoku RKG Whitelaw DD Murray M Hemorrhagic lesions resulting from *Trypanosoma vivax *infection in Ayrshire cattle Vet Parasitol 1989 31 187 197 10.1016/0304-4017(89)90069-1 2763442

[B52] Magona JW Walubengo J Odimin JT Acute haemorrhagic syndrome of bovine trypanosomosis in Uganda Acta Trop 2008 107 186 191 10.1016/j.actatropica.2008.05.019 18599006

[B53] Dickin SK Gibson WC Hybridisation with a repetitive DNA probe reveals the presence of small chromosomes in *Trypanosoma vivax* Mol Biochem Parasitol 1989 33 135 142 10.1016/0166-6851(89)90027-3 2725582

[B54] Masake RA Majiwa PAO Moloo SK Makau JM Njuguna JT Maina M Kabata J OleMoiYoi OK Nantulya VM Sensitive and specific detection of *Trypanosoma vivax *using the polymerase chain reaction Expt Para 1997 85 193 205 10.1006/expr.1996.4124 9030669

[B55] Morlais I Ravel S Grebaut P Dumas V Cuny G New molecular marker for *Trypanosoma (Duttonella) vivax *identification Acta Trop 2001 80 207 213 10.1016/S0001-706X(01)00160-7 11700177

[B56] Adams ER Hamilton PB Rodrigues AC Malele II Delespaux V Teixeira MMG Gibson WC New *Trypanosoma (Duttonella) vivax *genotypes from tsetse flies in East Africa Parasitology 2010 137 641 650 10.1017/S0031182009991508 19961657

[B57] Cortez AP Ventura RM Rodrigues AC Batista JS Paiva F Anez N Machado RZ Gibson WC Teixeira MMG The taxonomic and phylogenetic relationships of *Trypanosoma vivax *from South America and Africa Parasitology 2006 133 159 169 10.1017/S0031182006000254 16650339

[B58] Hoare CA The Trypanosomes of Mammals 1972 Oxford: Blackwell Scientific Publications

[B59] Wellde B Lotzsch R Deindl G Sadun E Williams J Warui G *Trypanosoma congolense*. I. Clinical observations of experimentally infected cattle Expt Para 1974 36 6 19 10.1016/0014-4894(74)90107-6 4846489

[B60] Hirumi H Hirumi K In vitro cultivation of *Trypanosoma congolense *bloodstream forms in the absence of feeder cell layers Parasitology 1991 102 225 236 10.1017/S0031182000062533 1852490

[B61] Majiwa PAO Matthyssens G Williams RO Hamers R Cloning and analysis of *Trypanosoma (Nannomonas) congolense *ILNAT 2.1 VSG gene Mol Biochem Parasitol 1985 6 97 108 2412117 10.1016/0166-6851(85)90052-0

[B62] Eshita Y Urakawa T Hirumi H Fish WR Majiwa PA Metacyclic form-specific variable surface glycoprotein-encoding genes of *Trypanosoma (Nannomonas) congolense* Gene 1992 113 139 148 10.1016/0378-1119(92)90389-7 1572537

[B63] Coustou V Guegan F Plazolles N Baltz T Complete in vitro life cycle of *Trypanosoma congolense*: Development of genetic tools PLoS Negl Trop Dis 2010 4 4 e618 20209144 10.1371/journal.pntd.0000618 PMC2830455

[B64] Peacock L Cook S Ferris V Bailey M Gibson W The life cycle of *Trypanosoma (Nannomonas) congolense *in the tsetse fly Parasites and Vectors 2012 in press 10.1186/1756-3305-5-109 PMC3384477 22676292

[B65] Gray MA Ross CA Taylor AM Luckins AG In vitro cultivation of *Trypanosoma congolense*: the production of infective metacyclic trypanosomes in cultures initiated from cloned stocks Acta Trop 1984 41 343 353 6152116

[B66] Helm JR Hertz-Fowler C Aslett M Berriman M Sanders M Quail MA Soares MB Bonaldo MF Sakurai T Inoue N Donelson JE Analysis of expressed sequence tags from the four main developmental stages of *Trypanosoma congolense* Mol Biochem Parasitol 2009 168 34 42 10.1016/j.molbiopara.2009.06.004 19559733 PMC2741298

[B67] Sakurai T Sugimoto C Inoue N Identification and molecular characterization of a novel stage-specific surface protein of *Trypanosoma congolense *epimastigotes Mol Biochem Parasitol 2008 161 1 11 10.1016/j.molbiopara.2008.05.003 18571746

[B68] Bayne RAL Kilbride EA Lainson A Tetley L Barry D A major surface antigen of procyclic stage *Trypanosoma congolense* Mol Biochem Parasitol 1993 61 295 310 10.1016/0166-6851(93)90075-9 8264732

[B69] Beecroft RP Roditi I Pearson TW Identification and characterization of an acidic major surface glycoprotein from procyclic stage *Trypanosoma congolense* Mol Biochem Parasitol 1993 61 285 294 10.1016/0166-6851(93)90074-8 7903427

[B70] Butikofer P Vassella E Boschung M Renggli CK Brun R Pearson TW Roditi I Glycosylphosphatidylinositol-anchored surface molecules of *Trypanosoma congolense *insect forms are developmentally regulated in the tsetse fly Mol Biochem Parasitol 2002 119 7 16 10.1016/S0166-6851(01)00382-6 11755181

[B71] Utz S Roditi I Renggli CK Almeida IC Acosta-Serrano A Butikofer P *Trypanosoma congolense *procyclins: Unmasking cryptic major surface glycoproteins in procyclic forms Euk Cell 2006 5 1430 1440 10.1128/EC.00067-06 PMC1539152 16896226

[B72] Downey N Donelson JE Expression of foreign proteins in *Trypanosoma congolense* Mol Biochem Parasitol 1999 104 39 53 10.1016/S0166-6851(99)00136-X 10589980

[B73] Downey N Donelson J Search for promotors for the *GARP *and rRNA genes of *Trypanosoma congolense* Mol Biochem Parasitol 1999 104 25 38 10.1016/S0166-6851(99)00135-8 10589979

[B74] Gibson W Species-specific probes for the identification of the African tsetse-transmitted trypanosomes Parasitology 2009 136 1501 1507 10.1017/S0031182009006179 19490726

[B75] Young CJ Godfrey DG Enzyme polymorphism and the distribution of *Trypanosoma congolense *isolates Ann Trop Med Parasit 1983 77 467 481 6660953 10.1080/00034983.1983.11811740

[B76] Gashumba JK Baker RD Godfrey DG *Trypanosoma congolense*: the distribution of enzymic variants in East and West Africa Parasitology 1988 96 475 486 10.1017/S0031182000080112 3405634

[B77] Masiga DK Smyth AJ Hayes PJ Bromidge TJ Gibson WC Sensitive detection of trypanosomes in tsetse flies by DNA amplification Int J Parasitol 1992 22 909 918 10.1016/0020-7519(92)90047-O 1459784

[B78] Majiwa PAO Masake RA Nantulya VN Hamers R Matthyssons G *Trypanosoma congolense*: identification of two karyotypic groups EMBO J 1985 4 3307 3313 4092683 10.1002/j.1460-2075.1985.tb04081.x PMC554658

[B79] Stevens JR Noyes H Dover GA Gibson WC The ancient and divergent origins of the human pathogenic trypanosomes, Trypanosoma brucei and T. cruzi Parasitology 1999 118 107 116 10.1017/S0031182098003473 10070668

[B80] Hamilton PB Stevens JR Gaunt MW Gidley J Gibson WC Trypanosomes are monophyletic: evidence from genes for glyceraldehyde phosphate dehydrogenase and small subunit ribosomal RNA Int J Parasitol 2004 34 1393 1404 10.1016/j.ijpara.2004.08.011 15542100

[B81] Gibson W Resolution of the species problem in African trypanosomes Int J Parasitol 2007 37 829 838 10.1016/j.ijpara.2007.03.002 17451719

